# Genetic Variant in Long Non-Coding RNA *H19* Modulates Its Expression and Predicts Renal Cell Carcinoma Susceptibility and Mortality

**DOI:** 10.3389/fonc.2020.00785

**Published:** 2020-05-20

**Authors:** Qiang Cao, Pengchao Li, Pu Cao, Jian Qian, Mulong Du, Li Li, Meilin Wang, Chao Qin, Pengfei Shao, Zhengdong Zhang, Qiang Lu, Zengjun Wang

**Affiliations:** ^1^Department of Urology, The First Affiliated Hospital of Nanjing Medical University, Nanjing, China; ^2^Department of Urology, The Second Hospital of Nanjing, Nanjing University of Chinese Medicine, Nanjing, China; ^3^Department of Molecular & Genetic Toxicology, Nanjing Medical University, Nanjing, China; ^4^Department of Ultrasound, The Second Hospital of Nanjing, Nanjing University of Chinese Medicine, Nanjing, China

**Keywords:** genetic variation, renal cell carcinoma, H19, susceptibility, mortality

## Abstract

The long non-coding RNA (lncRNA) H19 has been demonstrated to play a crucial role in carcinogenesis, including renal cell carcinoma (RCC). However, the impact of genetic variations in *H19* gene on RCC has not been investigated before. In the present study, we sought to evaluate whether genetic polymorphisms in *H19* are related to the susceptibility and mortality of RCC. We genotyped four widely studied polymorphisms in *H19* and assessed their relationship with susceptibility and prognosis of RCC in a case-control study compromising 1,027 cases and 1,094 controls. The functionality of the important polymorphism was further investigated by real-time polymerase chain reaction and luciferase reporter assay. We found that *H19* rs2839698 was significantly associated with risk and prognosis of RCC. Compared with the *H19* rs2839698 CC genotype, the variant genotypes (CT/TT) were significantly associated with increased risk of RCC (*P* = 0.023, OR = 1.21; 95% CI = 1.03–1.45). Besides, patients with variant genotypes (CT/TT) were more likely to develop large tumor (*P* = 0.003, OR = 1.47; 95% CI = 1.16–1.85) and advanced disease (*P* = 0.010, OR = 1.59; 95% CI = 1.12–2.26); and had a significantly unfavorable overall survival than those with the rs2839698 CC genotype (CT/TT vs. CC: Log-rank *P* = 0.026, HR = 2.25, 95%CI = 1.07–4.75). Furthermore, the CT/TT genotypes were associated with significantly increased expression of *H19* in renal tissue. The luciferase reporter assays revealed the potential effect of rs2839698 variant on the binding of microRNAs to *H19*. Our results suggest that the *H19* rs2839698 variant may be a genetic predictor of susceptibility and mortality of RCC. The risk effects and the functional impact of the variant on *H19* still need further validation.

## Introduction

Renal cell carcinoma (RCC) is the predominant type of kidney cancer, accounting for 5 and 3% of all malignancies in men and women, respectively (1, 2). It is estimated that up to 17% of patients show developed metastases at diagnosis and ~30% of the remaining patients will develop metastases even following surgical treatment (3, 4). Patients' prognosis is generally poor after detecting these metastases (3). Renal carcinogenesis is a complex process involving interactions between environmental and genetic factors (5). In recent years, genetic variations have been widely studied and demonstrated to influence the susceptibility, progression and prognosis of RCC (6–8).

Long non-coding RNAs (lncRNAs) are a class of endogenous cellular RNAs of more than 200 nucleotides in length that lack protein-coding capacity (9). Accumulating evidence suggests that lncRNAs play a crucial role in the occurrence and progression of various type of cancer (10, 11). LncRNA *H19* is a paternally imprinted gene which is located in chromosome 11p15.5 in human (12), and participates in numerous important biological and pathological processes through regulating the function of miRNAs (13) and mediating the DNA methylation (14). *H19* is closely linked to insulin-like growth factor-2 (*IGF2)* gene which is also located in the region subjected to imprinting by methylation and plays a crucial role in the normal growth and development of the fetus (15) and also important in cancer occurrence and progression (16). It has been suggested that aberrant *H19* expression was involved in variety of malignancies including bladder cancer (17), breast cancer (18), esophageal cancer (19), and RCC (20). In RCC, Wang et al. demonstrated that lncRNA H19 was over-expressed in tumor tissues and correlated with tumor stage, lymph node metastasis, and distant metastasis. Besides, the expression of lncRNA H19 was proposed to be an independent predictor for the clinical outcome of RCC patients (20).

Emerging studies have consistently demonstrated that genetic polymorphisms in *H19* gene are associated with the risk or prognosis of various cancers, including gastric cancer (21), colorectal cancer (22), bladder cancer (23, 24) and breast cancer (25). Most recently, a meta-analysis conducted by Hashemi et al. (26) on the association between *H19* polymorphisms and cancer risk has demonstrated that *H19* rs2839698 was associated with increased risk of gastrointestinal cancer. In light of the critical role of *H19* in RCC, it is possible that genetic variants in *H19* may have an effect on the risk and/or prognosis of RCC. However, to the best of our knowledge, no published study has yet investigated this issue. Therefore, in the present study, we selected four most widely studied polymorphisms in *H19* (rs2839698, rs3024270, rs217727, and rs2735971), and evaluated their associations with RCC risk and prognosis in a two-stage case-control study comprising a total of 1,027 cases and 1,094 controls in Chinese population.

## Materials and Methods

### Study Population

The present ongoing study was started in May 2004 and was approved by the Local Ethics Committees of the First Affiliated Hospital with Nanjing Medical University. The details of the inclusion criteria were described previously (7, 27). In brief, all subjects included in the study were ethnic Han Chinese individuals and genetically unrelated. Those patients who had received prior chemotherapy or radiotherapy, or had a different type of malignancy, were excluded from the present study. The controls were recruited from individuals who were seeking health examination at the outpatient department in the hospital and were frequency-matched to cases by sex and age (±5 years). Those subjects who were genetically related to the patients or had individual history of any cancer were excluded. The study was originally two-stage designed; the first set was recruited between May 2004 and October 2009 and a total of 355 RCC patients and 362 controls were included. The patients in this cohort were followed up prospectively every 6 months at the outpatient department or though patients' contacts. The endpoint was death or loss of follow-up which was considered censoring. Forty-four patients (12.4%) were excluded due to lack of adequate information for follow-up (41 cases) or low DNA quality (3 cases) for genotyping. The maximum follow-up time was 72 months and the median follow-up time was 25.1 months. The second set comprised of 672 RCC cases and 703 controls which was described previously (27). Before recruitment, a standard questionnaire was administered through face-to-face interviews by trained interviewers to obtain information on demographic data and related factors. The definition of smoker and drinker was described previously (28). At recruitment, information on demographic data and related factors of the participants were obtained by trained interviewers through face-to-face interviews. Each participant donated 5 ml venous blood collected in an EDTA tube after providing written informed consent at the interviews. In the present study, we genotyped and analyzed the four polymorphisms in the combination of both the set which comprised a total of 1,027 cases and 1,094 controls in the case-control analysis.

### Polymorphisms Selection and Genotyping

Genetic polymorphisms in both the *H19* gene and its promoter located in human chromosome 11p15.5 (position 2016406-2021693) were identified using the UCSC browser (http://genome.ucsc.edu/) with the selection criterion of a minor allele frequency (MAF) >0.05 in the CHB and JPT population from the 1000 Genomes Project. The detail information on selecting of these four polymorphisms was reported previously (24). Four tagged polymorphisms including rs2839698, rs217727, rs3741216, and rs3741219 were finally selected and investigated in this study. Genomic DNA was prepared and stored as described previously (7, 27). The genotyping of these four polymorphisms was performed using TaqMan SNP Genotyping Assays (Applied Biosystems, Foster City, CA, USA), as described previously (24). The sequences of the primers and probes are listed in Table S1.

### RNA Extraction and Quantitative Real-Time PCR (qPCR)

A total of 46 surgically removed renal cancer tissues with paired paratumor renal tissues (>2 cm away from tumor) were collected to test the expression level of lncRNA H19 *in vivo*. The tissues were immediately stored in liquid nitrogen after taken from the surgically removed samples. The RNA extraction and cDNA synthesis were described previously (29). The level of lncRNA H19 was measured by quantitative real-time reverse transcription (RT)-PCR on the ABI Prism 7900 sequence detection system (Applied Biosystems). β-actin was used as an internal reference gene. The primers used for lncRNA H19 were 5′-CCCACAACATGAAAGAAATGGTGC-3′(sense) and 5′-CACCTTCGAGAGCCGATTCC-3′(anti-sense), and the primers for β-actin were 5′-AGAAAATCTGGCACCACACC-3′(sense) and 5′-TAGCACAGCCTGGATAGCAA-3′(anti-sense). The reaction mixture contained 0.1M of each primer, 2 × SYBR Green PCR Master Mix (TaKaRa, Berkeley, CA, USA), and 1 mL of cDNA (1:10 dilution). The amplification was performed under the following conditions: 95°C for 30s, and 40 cycles of 95°C for 15s and 60°C for 30s. Each reaction was done in triplicate.

### Luciferase Reporter Assay

Reporter plasmids containing 160-bp *lncRNA H19* exon region fragments flanking the rs2839698 polymorphism with either the C or T allele were synthesized based on the reporter vector psiCHECK-2 (Promega, Madison, WI, USA). The accuracy of the constructed plasmids was verified by DNA sequencing. The renal cell adenocarcinoma cell line (786-o) and Human Embryonic Kidney 293 cells (HEK-293) were used for cell transfection and luciferase assays, as reported previously (29). Cells were seeded (at the density of 1.5 × 10^4^ cells/well) into 96-well plates containing culture medium, followed by 24-h incubation at the culture condition of 37°C, 100% humidity and 5% CO_2_. Using Lipofectamine 2000 reagent (Invitrogen, Carlsbad, CA, USA), reporter plasmids containing either rs2839698 C or T allele along with different miRNAs mimics or inhibitors were co-transfected into 786-o and HEK-293 cells. The non-miRNA-inserted plasmid was used as a negative control. Luciferase activity of the cells was measured with a Dual-Luciferase Reporter Assay System (Promega, Madison, WI, USA) 24 h after the transfection. Experiments were performed independently in triplicate for each of the plasmid constructs.

### Statistical Analysis

Student's *t*-test or chi-square test was used to test the differences in the distribution of demographic factors, clinical parameters, and frequencies of genotypes between cases and controls depending on the type of the variables. A goodness-of-fit chi-square test was used to test against departure from Hardy-Weinberg equilibrium in the control group for all the polymorphisms. False discovery rate (FDR) based on the Benjamini-Hochberg method was used to adjust the *P* value for multiple comparisons of the four SNPs before further analysis. The associations were considered statistically significant when FDR-adjusted *P* values were <0.05. The relationships between *H19* polymorphisms and RCC risk as well as tumor characteristics were assessed by computing odds ratios (ORs) and 95% confidence intervals (CIs) from unconditional logistic regression models adjusted for potential confounders. Survival time was calculated from the date of RCC diagnosis to the date of death or last follow-up. Specific survival curves according to clinical features and different *H19* genotypes were evaluated by using the Kaplan-Meier method and compared by the log-rank test. Cox regression analysis was used to determine predictive factors of RCC prognosis by estimating the hazard ratios (HRs) and their 95% CIs with adjustment for potential confounders. Differences in luciferase reporter activity and H19 expression levels among different groups were evaluated using Student's *t*-test or ANOVA. All analyses were performed using SAS 9.1.3 (SAS Institute, Cary, NC, USA) with two-sided *P* values. *P* value of <0.05 was considered statistically significant.

## Results

### Characteristics of RCC Patients and Controls

Frequency distributions of selected characteristics among the cases and controls are presented in Table S2. This is the same population as we described previously (27). Briefly, no significant difference was noticed between cases and controls regarding to age, gender, and drinking status (all *P* > 0.05). There were more smokers, hypertensive patients and diabetics in case group compared to that of the control group (*P* = 0.017, < 0.001 and <0.001, respectively). Among the patients, 65.3% of the patients were in stage I, whereas 19.5, 7.1, and 8.1% were found to have stage II, III, and IV diseases, respectively. The percentage of nuclear grade from I to IV was 21.6, 51.1, 20.7, and 6.5, respectively.

### Association of *H19* Polymorphisms With Risk of RCC

The associations between *H19* rs2839698, rs3024270, rs217727, and rs2735971 polymorphisms and risk of RCC are presented in Table 1. Genotype frequencies of the four polymorphisms in controls all conformed to HWE (all *P* > 0.05). As shown in Table 1, the genotypic distributions of rs2839698 in case and control groups was significantly different (*P* = 0.006), and remained significant after adjusting for multiple comparisons (FDR = 0.024). Compared with individuals carrying the rs2839698CC genotype, individuals harboring the rs2839698 variant TT genotype was associated with a significant increased RCC risk (*P* = 0.028, OR = 1.48, 95% CI = 1.16–2.42). Significant increased RCC risk was also noted when combining the CT and TT genotypes, and compared with rs2839698CC genotype (*P* = 0.023, OR = 1.21, 95%CI = 1.03–1.45). No significant evidence of association was found between any of the other polymorphisms and RCC risk. These results suggest that *H19* rs2839698 may confer individuals' genetic susceptibility to RCC.

**Table 1:** Genotype frequencies of the *H19* polymorphisms among the cases and controls and their associations with risk of RCC.

**Genotypes**	**Cases, n (%)**	**Controls, *n* (%)**	P^*^	**FDR^†^**	Adjusted OR^*^ (95%CI)
**rs2839698**					
CC	516 (50.2)	615 (56.2)	**0.006**	**0.024**	1.00 (reference)
CT	435 (42.4)	425 (38.9)	0.057		1.17 (0.98–1.41)
TT	76 (7.4)	54 (4.9)	**0.028**		**1.48 (1.16–2.42)**
CC	516 (50.2)	615 (56.2)			1.00 (reference)
CT/TT	511 (49.8)	479 (46.8)	**0.023**		**1.21 (1.03–1.45)**
**rs217727**					
CC	343 (31.4)	350 (34.1)	0.406	0.406	1.00 (reference)
CT	550 (50.2)	494 (48.1)	0.153		0.86 (0.71–1.06)
TT	201 (18.4)	183 (17.8)	0.391		0.89 (0.69–1.16)
CC	343 (31.4)	350 (34.1)			1.00 (reference)
CT+TT	751 (68.6)	677 (65.9)	0.155		0.87 (0.72–1.05)
**rs3741216**					
AA	791 (72.3)	728 (70.9)	0.260	0.520	1.00 (reference)
AT	255 (23.3)	264 (25.7)	0.247		1.12 (0.93–1.37)
TT	48 (4.4)	35 (3.4)	0.307		0.79 (0.57–1.24)
AA	791 (72.3)	728 (70.9)			1.00 (reference)
AT+TT	303 (27.7)	299 (29.1)	0.469		1.07 (0.89–1.30)
**rs3741219**					
TT	567 (51.8)	552 (53.8)	0.339	0.452	1.00 (reference)
TC	416 (38.0)	389 (37.9)	0.663		0.96 (0.80–1.15)
CC	111 (10.2)	86 (8.3)	0.142		0.79 (0.59–1.08)
TT	567 (51.8)	552 (53.8)			1.00 (reference)
TT+CC	527 (48.2)	475 (46.3)	0.376		0.97 (0.87–1.09)

* *Adjusted for age, sex, smoking, drinking status, diabetes and hypertension in logistic regression model. Bold-faced values indicate significant difference at 5% level*.

† *False discovery rate*.

### Effects of *H19* rs2839698 on Clinicopathological Characteristics of RCC Patients

To test whether the *H19* rs2839698 polymorphism could has effects on disease progression; we then analyze the association between rs2839698 genotypes and the tumor size, clinical stage and tumor grade of RCC in the patient group. As shown in Table 2, no significant association between rs2839698 genotypes and tumor grade was observed; however, the rs2839698 CT /TT genotypes were significantly more frequent in patients with tumor size more than 4 cm and those with advanced clinical stage (CT/TT vs. CC: *P* = 0.003, OR = 1.47; 95% CI = 1.16–1.85 and *P* = 0.010, OR = 1.59; 95% CI = 1.12–2.26, respectively). These results suggest that compare to patients with rs2839698 CC genotype, those harboring rs2839698 CT /TT genotypes were more prone to develop advanced disease. No significant association between any of the other polymorphisms and clinicopathological characteristics of RCC was found (data not show).

**Table 2:** Associations of *lncRNA H19* rs2839698 polymorphism with clinicopathological characteristics of RCC.

**Parameters**	**CC (n, %)**	**CT/TT (n, %)**	P^*^	Adjusted OR^*^ (95%CI)
**Tumor size**				
≤4	242 (50.3)	239 (49.7)	**0.003**	1.00 (reference)
>4	224 (41.0)	322 (59.0)		**1.47 (1.16–1.85)**
**Tumor grade**				
I+II	348 (46.6)	399 (53.4)	0.203	1.00 (reference)
III+IV	118 (42.1)	162 (57.9)		1.21 (0.93–1.61)
**Clinical stage**				
I+II	410 (47.1)	461 (52.9)	**0.010**	1.00 (reference)
III+IV	56 (35.9)	100 (64.1)		**1.59 (1.12–2.26)**

* *Adjusted for age, sex, smoking, drinking status, diabetes, and hypertension in logistic regression model. Bold-faced values indicate significant difference at 5% level*.

### Effects of *H19* rs2839698 on RCC Patients' Survival

**Figure 1:**
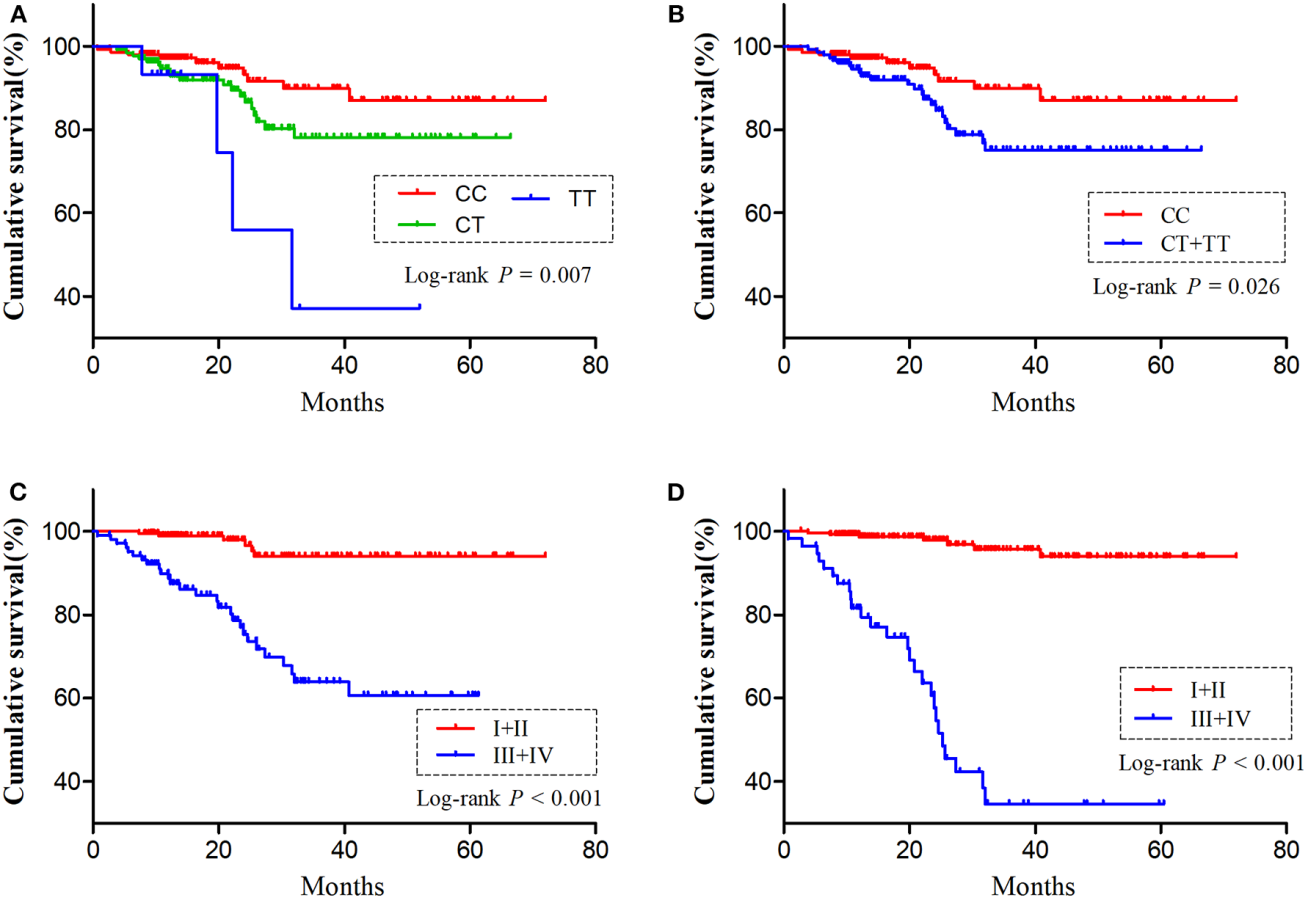
The influence of *H19* rs2839698 polymorphism on RCC survival. Kaplan-Meier survival curves illustrate RCC overall survival according to **(A,B)** different *H19* rs2839698 genotypes, **(C)** tumor grade (I + II vs. III + IV) and **(D)** clinical stage (I + II vs. III + IV).

As *H19* rs2839698 was shown to contribute to RCC progression, we then sought to explore the association between rs2839698 polymorphism and survival of RCC patients using the Log-rank test and Kaplan-Meier analysis. As shown in Table 3 and Figures 1A,B, we found that the rs2839698 polymorphism was significantly associated with patients' survival (Log-Rank *P* = 0.006). The association remained significant after adjusting for multiple comparisons (FDR = 0.024). Compared with patients harboring the rs2839698CC genotype, those patients with rs2839698TT or CT/TT genotypes had an unfavorable RCC survival (HR = 4.29, 95%CI = 1.15–15.94 and HR=2.25, 95% CI=1.07–4.75, respectively). However, no significant evidence of association was found between the other polymorphisms and RCC survival as shown in Table 3. We then performed a univariate and multivariate Cox proportional hazard analysis for survival of RCC patients. As shown in Table 4 and Figures 1C,D, clinical stage, tumor grade, and *H19* rs2839698 (CT/TT vs. CC) were associated with RCC survival in univariate analysis; in multivariate analysis, clinical stage was found to be the best prognostic factor for RCC survival, followed by tumor grade (*P* < 0.001, HR = 15.5; 95% CI = 5.94–40.34 and *P* = 0.004, HR = 3.98; 95% CI = 1.55–10.23, respectively). Interestingly, *H19* rs2839698 (CT/TT vs. CC) was also an independent predictor of RCC survival (*P* = 0.33, HR = 2.25; 95% CI = 1.07–4.75) in the multivariate analysis.

**Table 3:** Associations of *H19* polymorphisms with RCC patients' survival.

**Polymorphisms**	**Patients (*N* = 311)**	**Deaths (*N* = 33)**	5-yr survival^*^ (%)	**Log-Rank *P***	**FDR^**#**^**	**HR (95% CI)^†^**
**rs2839698**						
CC	158	10	87.1%	**0.006**	**0.024**	1.00 (reference)
CT	138	19	78.3%			1.88 (0.80–4.47)
TT	15	4	37.3%			**4.29** **(1.15–15.94)**
CC	158	10	87.1%	**0.026**		1.00 (reference)
CT+TT	153	23	75.2%			**2.25** **(1.07–4.75)**
**rs3741216**						
AA	188	23	80.5%	0.830	0.830	1.00 (reference)
AT	79	8	81.4%			1.07 (0.47–2.44)
TT	11	2	83.9%			1.97 (0.45–8.72)
AA	188	23	80.5%	0.885		1.00 (reference)
AT+TT	90	10	82.1%			1.17 (0.55–2.52)
**rs3741219**						
TT	149	22	79.1%	0.212	0.424	1.00 (reference)
TC	101	10	82.1%			0.61 (0.29–1.30)
CC	28	1	90%			0.25 (0.03–1.02)
TT	149	22	79.1%	0.102		1.00 (reference)
TT+CC	129	11	83.5%			0.54 (0.26–1.13)
**rs217727**						
CC	103	16	78.7%	0.415	0.553	1.00 (reference)
CT	133	12	85.4%			0.62 (0.29–1.34)
TT	42	5	78.5%			0.82 (0.30–2.27)
CC	103	16	78.7%	0.209		1.00 (reference)
CT+TT	175	17	82.2%			0.67 (0.33–1.35)

*HR, hazards ratio; CI, confidence interval*.

* *Proportion of survival derived from Kaplan-Meier analysis*.

† *Adjusted for age, gender, smoking, drinking status, diabetes, and hypertension as well as tumor grade and clinic stage*.

# *False discovery rate*.

**Table 4:** Univariate and multivariate Cox proportional hazard analysis of death risk in patients with RCC.

**Parameters**	**Univariate**		**Multivariate**^**†**^	
	HR (95% CI)^*^	***P* value**	HR (95% CI)^*^	***P* value**
Age (≤ 56 vs. >56)	1.68 (0.83–3.37)	0.147		
Gender (female vs. male)	0.89 (0.44–1.82)	0.758		
Smoking status (never vs. ever)	0.97 (0.47–2.00)	0.929		
Drinking status (never vs. ever)	1.33 (0.63–2.80)	0.458		
Diabetes (no vs. yes)	0.66 (0.20–2.16)	0.493		
Hypertension (no vs. yes)	1.53 (0.77–3.05)	0.222		
Tumor grade (III/IV vs. I/II)	**8.88 (3.66–21.52)**	**<0.001**	**3.98 (1.55–10.23)**	**0.004**
Clinical stage (III/IV vs. I/II)	**20.28 (8.78–46.87)**	**<0.001**	**15.5 (5.94–40.34)**	**<0.001**
*H19 rs2839698* (CT/TT vs. CC)	**2.27 (1.08–4.77)**	**0.030**	**2.25 (1.07–4.75)**	**0.033**

* *HR, hazards ratio; CI, confidence interval*.

*In this multivariate analysis age, gender, smoking, drinking status, diabetes, hypertension, tumor stage, clinic stage, and the number of variant alleles were included. Bold-faced values indicate significant difference at 5% level*.

### Expression of *H19* in RCC and the Influence of rs2839698 Polymorphism on *H19* Expression

**Figure 2:**
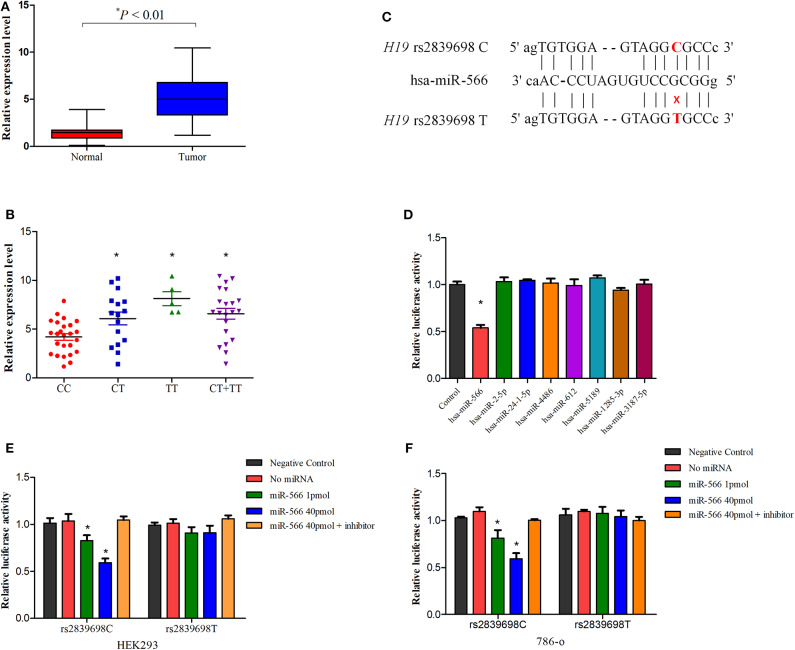
The influence of *H19* rs2839698 variant on H19 expression. **(A)** Distribution and comparison of *H19* expression in tumors and adjacent normal tissues (*P* < 0.01); **(B)** Association between the *H19* expression in renal tissues and *H19* rs2839698 genotypes (*P* = 0.019, 0.004, and 0.001 for CT vs. CC, TT vs. CC and CT/TT vs. CC, respectively); **(C)** Schematic image of binding interaction between miR-566 and *H19* rs2839698 C and T alleles. **(D)** The psi-CHECK-2-H19-C-allele and miRNA mimics were co-transfected into HEK293 cell line. Relative luciferase activities in the cells were measured from three independently differentiated clones in each genotype. Only the luciferase activity in cells co-transfected with miR-566 was significantly decreased (*P < 0.05). The psi-CHECK-2-H19-C-allele or T allele construct as well as miR-566 mimic and inhibitor were co-transfected into **(E)** HEK293 and **(F)** 786-0 cell lines. *P < 0.05, compared with the psi-CHECK-2-rs2839698-C/T allele constructs co-transfected with miRNA control.

To further explore the potential functionality of *H19* rs2839698, we investigated the effect of this polymorphism on *H19* expression in cancer tissues and paratumor normal renal tissues using real-time quantitative PCR. As shown in Figure 2A, the *lncRNA H19* expression level in tumor tissues was significantly higher than that in the adjacent normal tissues (*P* < 0.01) which was consistent with previous reports (20, 30). Besides, in normal tissues, as shown in Figure 2B, compared with individuals carrying the rs2839698CC genotype, individuals carrying the variant T allele (CT/TT genotypes) had increased levels of *H19* expression (*P* = 0.019, 0.004 and < 0.001 for CT vs. CC, TT vs. CC and CT/TT vs. CC respectively). These results suggested that overexpression of lncRNA H19 may contribute to renal carcinogenesis and that rs2839698 may be involved in the process by regulating the expression levels of lncRNA H19.

### Functional Characterization of lncRNA H19 rs2839698 Polymorphism

An *in silico* analyses was performed previously using RNAfold and SNPfold databases to explore whether biological functions of *H19* would notably affected by the genetic variants (22). As reported previously, the secondary structure *H19* was altered by rs2839698 variant (22). As rs2839698 is located in the 3′untranslated region of *H19* gene, we speculate that the increased expression of H19 could be associated with alteration of miRNAs target. As predicted by bioinformatics mode (22), the change from a C allele to T allele at rs2839698 may disrupt the binding of hsa-miR-24-1-5p, hsa-miR-4486, hsa-miR-566, and hsa-miR-24-2-5p, and create binding site for hsa-miR-612, hsa-miR-5189, hsa-miR-1285-3p and hsa-miR-3187-5p. To test the prediction, we then introduced the luciferase reporter vectors (rs2839698 C or T allele) together with the mimics of the miRNAs into HEK-293 cell line. As shown in Figures 2C,D, in the presence of C allele, the luciferase activity of cells co-transfected with miR-566 was significantly decreased compared with the control in HEK-293 cells. No significant differences of the luciferase activity were observed for all the miRNAs in the presence of T allele in HEK-293 cells, as shown in Figure S1. To further confirm the prediction, the reporter vectors (psiCHECK-2-rs2839698C and psiCHECK-2- rs2839698T), miR-556 mimics and inhibitor were transiently co-transfected into 786-O and HEK-293 cell lines. In both cell lines, as shown in Figures 2E,F, luciferase activity was significant decreased in the presence of C allele when vectors co-transfected with miR-556 mimics; however, no significant alternations in luciferase activity were observed in the presence of the miR-556 inhibitor. No significant influence of miR-566 inhibitor alone on the luciferase activity of H19 rs2839698 variant, as shown in Figure S2.

## Discussion

In the preset study, we investigated the associations between polymorphisms in *H19* and RCC susceptibility as well as prognosis in a Chinese population. Our results suggested that the *H19* rs2839698 variant was associated with an increased risk of RCC. We also demonstrated that this variant was associated with larger tumor and advanced clinical stage of RCC, and was an independent prognostic predictor of patients' survival, along with clinical tumor stage and grade in multivariate analysis. The functional role of rs2839698 variant in elevating the *H19* level was subsequently demonstrated in *in vivo* and *in vitro* assays. To the best of our knowledge, this is the first study to demonstrate a role of *H19* rs2839698 variant in the etiology and prognosis of RCC.

Long non-coding RNA *H19* is a paternally imprinted oncofetal gene located on chromosome 11p15.5 and acts as an oncogene involving in the process of occurrence and metastasis of malignancies (31, 32). Highly up-regulated of *H19* was demonstrated in a variety of tumors including colorectal cancer (33), hepatocellular cancer (34), breast cancer (35), lung cancers (36), and RCC (20, 30). In RCC, Wang et al. demonstrated that relative level of H19 was significantly higher in renal tumors compared to the adjacent normal renal tissues. Besides, patients with higher H19 expression had more advanced clinical stage and poorer prognosis than those with lower expression (20). Consistent with this study, He et al. also showed that H19 was overexpressed in renal carcinoma and further demonstrated H19 was involved in the migration and invasion of RCC through miR-29a-3p/E2F1 pathway (30). In the present study, we also observed that the *H19* level was significantly higher in RCC tissues than in normal renal tissues, which further confirmed a causative role of the H19 in RCC.

Accumulation of evidence has suggested that genetic variation in lncRNAs could play a crucial role in cancer susceptibility and prognosis. Given the important role of *H19* in tumorigenesis, the influence of genetic polymorphisms in *H19* on caner susceptibility and prognosis has been extensively investigated in various malignancies by previous studies (21–25). Consistent with our results, several other studies have also demonstrated *H19* rs2839698 variant to be a risk factor for gastrointestinal cancer (26), colorectal cancer (22), and hepatocellular cancer (37). In hepatocellular cancer, Yang and colleagues found that *H19* rs2839698 variant genotype not only confer an increased HCC risk, but also a potential genetic predictor for HCC prognosis in the subgroup of ever smokers (37). Similar to our findings, they found that patients with *H19* rs2839698 variant genotype (CT) had a significant unfavorable prognosis (*P* = 0.035, HR = 5.19, 95% CI = 1.12–24.07).

Accumulating evidence has shown that genetic variations could exert function through altering both DNA and RNA secondary structures (38, 39), and then influencing the binding affinity of miRNAs to its' specific region (40, 41). As for lncRNAs, Zheng et al. demonstrated that a G>A change at rs11655237 in LINC00673 could create a target binding site for miR-1231, which diminished the effect of LINC00673 in an allele-specific manner and thus confers susceptibility to pancreatic cancer (42). Recently, in colorectal cancer, Wu et al. showed that the allele C>G change at rs664589 in MALAT1 altered the binding affinity of the miR-195-5p to the mutant region leading to increased MALAT1 expression and then promoting the colorectal cancer growth, and metastasis (43). However, to the best of our knowledge, no published studies have focused on the effect of *H19* rs2839698 in regulating the *H19* expression and function.

As predicted by *in silico* analyses using the SNPfold algorithm, the secondary structure of *H19* was dramatically changed with rs2839698 C/T alleles, suggesting the rs2839698 variant might affect the susceptibility and prognosis of RCC by altering the secondary structure *H19*. It has been suggested that lncRNAs and miRNAs could negatively modulated each other by forming a reciprocal repression-regulatory loop (44, 45). For instance, Hirata et al. found that the interaction between MALAT1 and miRNA-205 had reciprocal effects in RCC (44). Using bioinformatics analysis and luciferase reporter analysis, we identified that hsa-miR-566 might lose the target in *H19* in the presence of rs2839698 T allele. Interestingly, hsa-miR-566 was demonstrated to be an oncogene and a potential biomarker for prognosis in renal cell carcinoma (46). Therefore, we speculate that the rs2839698 T allele might block the binding of hsa-miR-566 to the 3'UTR of *H19* and then disrupt the reciprocal repression-regulatory loop between hsa-miR-566 and *H19*, and subsequently, lead to up regulation of *H19*. This is also consistent with our findings that individuals with the rs2839698 TT genotype showed increased *H19* level compared to individuals with the rs2839698 CC genotype. Given the oncogene role of *H19*, up-regulation of *H19* due to the rs2839698 variant genotype may confer RCC susceptibility and prognosis, which may explain our findings in the case-control study. However, limitations should be concerned when interpreting our results. First, the present case-control study was hospital-based instead of population-based; therefore the possibility of the selection bias of individuals associated with a particular genotype could not be rule out. Besides, our functional results toward the *H19* rs2839698 variant are only preliminary, the exact mechanism underlying the functional effects of this variant on *H19* expression still requires further investigation.

In conclusion, this is the first study demonstrating a role of *H19* rs2839698 polymorphism in RCC susceptibility and prognosis in a Chinese cohort. Our results, although preliminary, highlight a putatively functional effect of this variant on regulating the *H19* expression, which might through disrupting the reciprocal repression-regulatory loop between hsa-miR-566 and *H19*. However, further validation in a larger population and functional studies are still warranted.

## Data Availability Statement

All datasets generated for this study are included in the article/Supplementary Material.

## Ethics Statement

The studies involving human participants were reviewed and approved by the Local Ethics Committees of the First Affiliated Hospital with Nanjing Medical University. The patients/participants provided their written informed consent to participate in this study.

## Author Contributions

QC and PL proposed the hypothesis and designed the experiments. QC, JQ, MD, and CQ performed the experimental work and manuscript drafting. PS, MW, ZZ, and LL performed the analysis and manuscript drafting. PC, QC, and PL performed the experimental work. QL, ZW, and ZZ conceived the study, participated in its design and coordination, and helped edit the manuscript.

## Conflict of Interest

The authors declare that the research was conducted in the absence of any commercial or financial relationships that could be construed as a potential conflict of interest.
