# Supplementary material for: Genetic Variant in Long Non-Coding RNA H19 Modulates Its Expression and Predicts Renal Cell Carcinoma Susceptibility and Mortality
Source: Front Oncol. 2020 May 20;10:785. doi: 10.3389/fonc.2020.00785 (PMC7251175; doi:10.3389/fonc.2020.00785)
Supplement: Supplementary file 1 [file Data_Sheet_1.docx]

| Polymorphisms | Primer sequence (5’-3’) | Probe sequence (MGB) |
| --- | --- | --- |
| rs2839698 C>T | F:CATCGTCCCCAGCTGATGTC | C: FAM-CTGGGCGCCTACT |
|  | R: GGAGTGATGACGGGTGGAG | T: HEX-CCTGGGCACCTAC |
| rs3741219 T>C | F: CGAGTGTGCGTGAGTGTGAG | T: HEX-AAGTGCCTGTGCAGG |
|  | R: TAATGGAATGCTTGAAGGCTGCTC | C: FAM-AGTGCCTGCGCAGG |
| rs217727 C>T | F: CAAAGAGACAGAAGGATGAAAAAGAA | C: FAM-TCAACCGTCCGCCG |
|  | R: CGGCGACTCCATCTTCATG | T: HEX-TCAACCGTCCACCG |
| rs3741216 A>T | F: GCCTCCACGACTCTGTTTCC | A: HEX-CCCTTCTGAATTTAA |
|  | R: CACAACTCCAACCAGTGCAAA | T: FAM-CCCTTCTGAATTTTAT |

**Table S1, Information of primers and probes for the selected polymorphisms**

**Table S2** Distribution of selected variables between the renal cell carcinoma cases and control subjects.

| Variables | Case (N, %) | Controls (N, %) | *P* |
| --- | --- | --- | --- |
|  | 1027 | 1094 |  |
| Age (mean ± SD) | 56.7 ± 12.1 | 57.1±12.1 | 0.522 |
| Gender |  |  |  |
| Male | 652 (63.5) | 704 (64.4) | 0.678 |
| Female | 375 (36.3) | 390 (35.6) |  |
| Smoking status |  |  |  |
| Never | 647 (63.0) | 743 (67.9) | **0.017** |
| Former | 380 (37.0) | 351 (32.1) |  |
| Drinking status |  |  |  |
| Never | 750 (73.0) | 807 (73.8) | 0.701 |
| Ever | 277 (27.0) | 287 (26.2) |  |
| Hypertension |  |  |  |
| No | 626 (70.0) | 810 (74.0) | **<0.001** |
| Yes | 401 (30.0) | 284 (26.0) |  |
| Diabetes |  |  |  |
| No | 895 (87.2) | 1027 (93.9) | **<0.001** |
| Yes | 132 (12.8) | 67 (6.1) |  |
| Clinical stage |  |  |  |
| I | 671 (65.3) |  |  |
| II | 200 (19.5) |  |  |
| III | 73 (7.1) |  |  |
| IV | 83 (8.1) |  |  |
| Grade |  |  |  |
| I | 222 (21.6) |  |  |
| II | 525 (51.1) |  |  |
| III | 213 (20.7) |  |  |
| IV | 67 (6.5) |  |  |

^a^ Student’s t-test for age distributions between cases and controls; two-sided χ2-test for others selected variables between cases and controls.


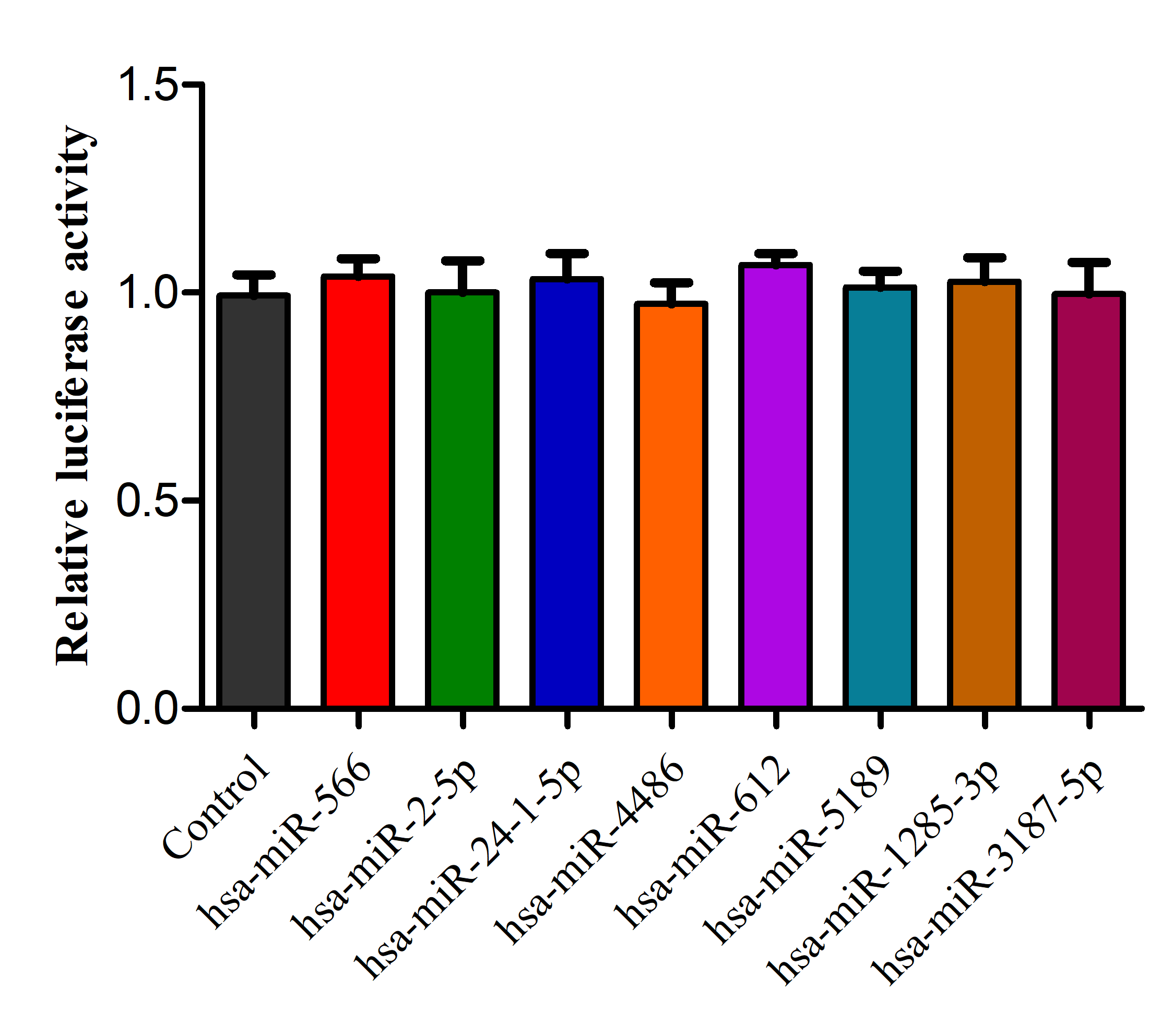


**Figure S1.** The psi-CHECK-2-H19-T-allele and miRNA mimics were co-transfected into HEK293 cell line. Relative luciferase activities in the cells were measured from three independently differentiated clones in each genotype. No significant alteration was measured.

**Figure S2.** The influence of miR-566 inhibitor alone on the luciferase activity of H19 rs2839698 variant. No significant difference was observed.
